# Long-Term Effects of Low-Intensity Blast Non-Inertial Brain Injury on Anxiety-Like Behaviors in Mice: Home-Cage Monitoring Assessments

**DOI:** 10.1089/neur.2021.0063

**Published:** 2022-01-11

**Authors:** Heather R. Siedhoff, Shanyan Chen, Ashley Balderrama, Grace Y. Sun, Bastijn Koopmans, Ralph G. DePalma, Jiankun Cui, Zezong Gu

**Affiliations:** ^1^ Harry S. Truman Memorial Veterans' Hospital Research Service, Columbia, Missouri, USA.; ^2^ Department of Pathology and Anatomical Sciences, University of Missouri School of Medicine, Columbia, Missouri, USA.; ^3^ Department of Biochemistry, University of Missouri School of Medicine, Columbia, Missouri, USA.; ^4^ Sylics (Synaptologics BV), Bilthoven, The Netherlands.; ^5^ Office of Research and Development, Department of Veterans Affairs, Washington, DC, USA; Department of Surgery, Uniformed Services University of the Health Sciences, Bethesda, Maryland, USA.

**Keywords:** anxiety-like behaviors, home-cage monitoring, primary open-field blast, spontaneous activity

## Abstract

Mild traumatic brain injury induced by low-intensity blast (LIB) exposure poses concerns in military personnel. Using an open-field, non-inertial blast model and assessments by conventional behavioral tests, our previous studies revealed early-phase anxiety-like behaviors in LIB-exposed mice. However, the impact of LIB upon long-term anxiety-like behaviors requires clarification. This study applied a highly sensitive automated home-cage monitoring (HCM) system, which minimized human intervention and environmental changes, to assess anxiety-like responses in mice 3 months after LIB exposure. Initial assessment of 72-h spontaneous activities in a natural cage condition over multiple light and dark phases showed altered sheltering behaviors. LIB-exposed mice exhibited a subtle, but significantly decreased, duration of short shelter visits as compared to sham controls. Other measured responses between LIB-exposed mice and sham controls were insignificant. When behavioral assessments were performed in a challenged condition using an aversive spotlight, LIB-exposed mice demonstrated a significantly higher frequency of movements of shorter distance and duration per movement. Taken together, these findings demonstrated the presence of chronic anxiety-like behaviors assessed by the HCM system under both natural and challenged conditions in mice occurring post-LIB exposure. This model thus provides a platform to test for screening and interventions on anxiety disorders occurring after LIB non-inertial brain injury.

## Introduction

Mild traumatic brain injury (mTBI) caused by non-inertial exposure to low-intensity blast (LIB)—entailing elimination of head movement—in an open-field environment results in neurobehavioral and pathological alterations.^1,2^ In military populations, >82% of TBIs reported from 2000 to 2021 were mTBIs.^3^ LIB exposures experienced during combat operations and occupational training are a primary cause of military-obtained mTBIs.^4,5^

Among the prominent comorbidities of blast-induced mTBIs, post-traumatic stress disorder (PTSD) and anxiety highly deteriorate the quality of life.^6^ Veterans of the first Gulf War reported >2 times higher prevalence of anxiety disorders.^7^ Progressive and accumulative clinical neuropsychiatric abnormalities, such as post-concussive syndrome, may not be detected until months or years after blast exposure.^8–10^

Primary blast injury occurs by energy transfer of supersonic primary blast waves generated by high-energy explosives.^11–13^ In previous studies, we implemented a militarily realistic open-field LIB injury model where mice were exposed with a magnitude of a 46.6-kPa peak overpressure and a maximum impulse of 60.0 kPa*ms without detectable acceleration effects.^1,14–18^ Thus, the resulting behavioral, pathophysiological, and biochemical alternations of the LIB injury were, in all likelihood, attributable to the primary blast effects, but not inertial brain injury.^1,14–18^ We identified anxiety-like behaviors post-LIB exposure as indicated by open-field and light-dark box behavioral tests up to 7 days post-injury.^1^ Ultrastructural abnormalities of mitochondria, myelinated axons, and asymmetrical synapses, as well as increased mitochondrial fission, oxidative stress, compromised mitophagy, synaptic dysregulation, and neurodegeneration, were detected in brains of LIB-exposed mice up to 1 month.^1,16–18^

It is crucial to extend such studies by implementation of novel assessments on multi-domains of neurological functions. The common use of conventional behavioral assessments involves many impeding factors, including insensitivity, circadian-dependent rodent behaviors, confounding factors by human handling, and environment-induced stress. Therefore, incorporating a home-cage monitoring (HCM) system with specific features can overcome the limitations and enable more prolonged periods of investigation.^19–23^ In addition, the HCM system provides ability to collect extensive data spanning a wide range of parameters.^24^

This study used the HCM system to evaluate chronic-phase behavioral alterations in LIB-exposed mice under natural and challenged conditions. Our results showed that a single LIB exposure induced an elevated level of anxiety-like behaviors as compared to sham controls in both spontaneous and affected situations. This research provides a platform of diagnostic testing and possible interventions on anxiety disorders for clinical translation on combat-related mTBI.

## Methods

### Animals and open-field blast setting

All experiments were performed in a randomized-blinded manner and in accordance with the University of Missouri–approved protocols for the Care and Use of Laboratory Animals and the Animal Research: Reporting of In Vivo Experiments (ARRIVE) guidelines. This study involved 52 C57BL/6J male mice (2 months old; The Jackson Laboratory, Bar Harbor, ME) housed in groups with a 12-h light/dark cycle (lights on/off at 7:00 am/7:00 pm) in standard mouse cages containing bedding with food and water provided *ad libitum*. Mice were housed individually during HCM behavioral tests and returned to their original cages after tests. Open-field LIB exposure was conducted at the open-air blast quarry at the Missouri University of Science and Technology, as previously reported.^1,2,16,17^

Mice were assigned randomly into two groups: LIB-exposed mice and sham controls. Mice were anesthetized with an intraperitoneal injection of 10 μL/g body weight of ketamine/xylazine mixture (12.5 mg/mL of ketamine and 0.625 mg/mL of xylazine) and moved to the blast field within 30 min. Mice in the sham control group underwent the identical anesthesia procedures, but without LIB exposure. Mice were placed in metal-mesh holders in the prone position. Animal holders were fixed on 1-m-height platforms, which were a 3-m distance away from a 350-g sphered C4 generating a magnitude of 46.6 kPa peak overpressure, a maximum impulse of 60.0 kPa*ms, as described previously.^1,2,16,17^ Animals did not experience head or body motion attributable to the blast. Mice were monitored and allowed access to food and water *ad libitum*.

### Testing spontaneous behaviors in a home-cage environment

**FIG. 1.**
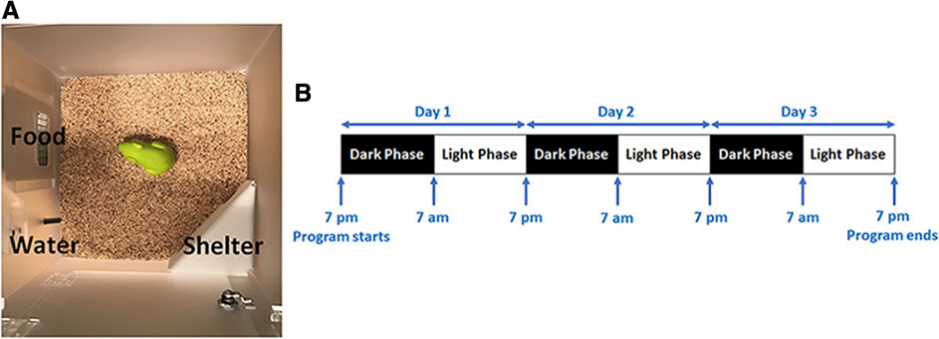
Experimental setup and timeline of the spontaneous HCM system. (**A**) Experimental setup of the spontaneous behavioral tests in the HCM system. (**B**) Timeline of the spontaneous behavioral tests in the HCM system. HCM, home-cage monitoring.

Spontaneous behaviors were assessed in sham controls (*n* = 23) and LIB-exposed (*n* = 29) mice at 3 months post-LIB exposure with the PhenoTyper home cages (Model 3000; Noldus Information Technology, Wageningen, The Netherlands), as previously described.^19,25,26^ Briefly, home cages (L = 30 × W = 30 × H = 35 cm) were composed of a top control unit, four semitransparent Perspex walls, an opaque Perspex floor, a shelter, and food and water stations. Mice were housed individually with bedding (Bed-o'Cobs^®^, Laboratory Animal Bedding; The Andersons Laboratory Animal Bedding, Maumee, OH) and provided food and water *ad libitum* (Fig. 1A). Mouse behaviors were automatically recorded through a 24/7, infrared-sensitive automated video-tracking system controlled by EthoVision XT software (v14; Noldus Information Technology) sampling at a rate of 15 fps. The experiment started at 7:00 pm for 72 h, covering three cycles of dark and light phases (Fig. 1B).

Data were uploaded to the Web-based AHCODA-DB (Sylics, Bilthoven, The Netherlands) for meta-analysis. The spontaneous behavioral parameters in six categories were activity bouts, kinematics, sheltering, dark-light index, habituation, and light/dark phase transition.^19,25,26^ Briefly, movements were classified into *short movements*, as turning or rearing against the wall, and *long movements*, as traveling from one location to the next. *Activity bouts* stopped at the encounter of a long arrest segment or a *shelter visit* that exceeded the short shelter visit threshold. *Habituation* and *light/dark index* were used to detect pronounced behaviors during the light or dark phases and early or late stages of the tests. *Habituation indices* were calculated as ratios of values on day 3 / values on day 1, measured in 12-h time bins. *Dark-light indices* were calculated as ratios of dark value / (dark value + light value) on the third day.

Frequency distributions of sheltering behaviors were further classified as fitting into three Gaussian curves. *Short shelter visits* were distinguished as visit duration within the 90th percentile of the first Gaussian curve, representing in-and-out of the shelter during bouts of activity (for seconds). *Long shelter visits* were distinguished as visit duration within the third Gaussian curve representing resting or sleeping within the shelter (for hours). *Light/dark phase transition* patterns were analyzed with proportion changes of activity time in the few hours preceding and following the light/dark phase shifts.

### Testing behaviors in response to aversive spotlight stimulation in a home-cage environment (LightSpot test)

In order to investigate the responses of mice to a mild aversive stimulus, a yellow light-emitting diode light (2000 lux, comparable to the light of a typical overcast day in the absence of heat production) was introduced without generating explicit pain or discomfort to sham controls (*n* = 20) and LIB-exposed (*n* = 28) mice.^27^ The LightSpot test processed pre-calibrated experimental protocols in the absence of human intervention. The spotlight was automatically switched on at 7:15 pm, 15 min after the acquisition in the dark phase (started at 7:00 pm). The spotlight shined on the upper left corner of the unit, aiming a light beam at the feeding station. The spotlight persisted on for 1 h and was switched off at 8:15 pm.

### Data and statistical analyses

Data were analyzed using Prism software (GraphPad Software Inc., La Jolla, CA). An unpaired one-tailed Student's *t*-test was performed for any two-group comparisons based on predictions formulated with earlier data. Two-way repeated-measures analysis of variance (ANOVA) and Bonferroni multiple comparisons were performed for assessing 72-h spontaneous behaviors consecutively of distance moved, duration of arrests, duration in feeding zone, and duration in spout zone, as well as sheltering behaviors in response to spotlight exposure. Data are presented as mean values ± standard error of the mean. Difference was considered significant at *p* < 0.05 for all analysis.

## Results

### Low-intensity blast–exposed mice demonstrated long-term alterations of sheltering behaviors assessed in a home-cage environment

**FIG. 2.**
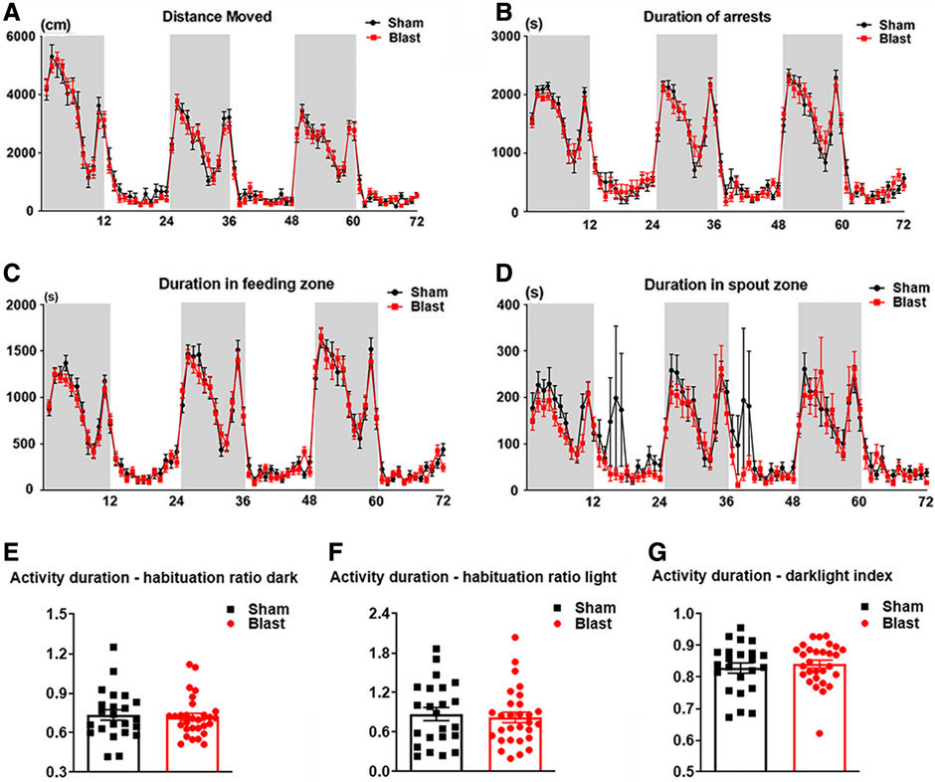
General behavioral tests in the spontaneous HCM system. Spontaneous behaviors are assessed consecutively for three dark phases (shaded regions of graph) and three light phases (unshaded regions of graph), including (**A**) distance moved, (**B**) duration of arrests, (**C**) duration in feeding zone, and (**D**) duration in spout zone. Data were analyzed by two-way repeated-measures ANOVA and Bonferroni multiple comparisons. (**E,F**) Habituation ratios of activity duration during (**E**) dark and (**F**) light phases, as well as (**G**) dark-light index of activity duration are analyzed by the one-tailed, unpaired Student's *t*-test. Data are presented as means ± SEM. *n* = 23 sham mice and *n* = 29 LIB-exposed mice. ANOVA, analysis of variance; HCM, home-cage monitoring; LIB, low-intensity blast; SEM, standard error of the mean.

In order to detect the presence of long-term behavioral impairments after LIB exposure, this study used the automated high-throughput HCM system and Web-based AHCODA-DB bioinformatics analysis. Spontaneous behaviors were assessed in LIB-exposed mice and sham controls for 72 h (consecutive). All mice showed increased activity during the first 5–6 h, demonstrating an exploratory behavior in a new environment (Fig. 2A). Two-way repeated-measures ANOVA showed that there were no significant effects of LIB exposure in distance moved (*p* = 0.1820), arrest duration (*p* = 0.7955), and duration in feeding zone (*p* = 0.2417; Fig. 2A–C). Duration in spout zone (Fig. 2D) was significantly longer in sham controls compared to LIB-exposed mice, (*p* = 0.0002), though with large variations. Habituation ratios of activity duration showed no significant difference between LIB-exposed mice and sham controls during light and dark phases (*p* = 0.3412 in light phases; 0.3739 in dark phases; Fig. 2E,F). In addition, no significant difference of dark-light index was found between LIB-exposed mice and sham controls (*p* = 0.2696; Fig. 2G).

**FIG. 3.**
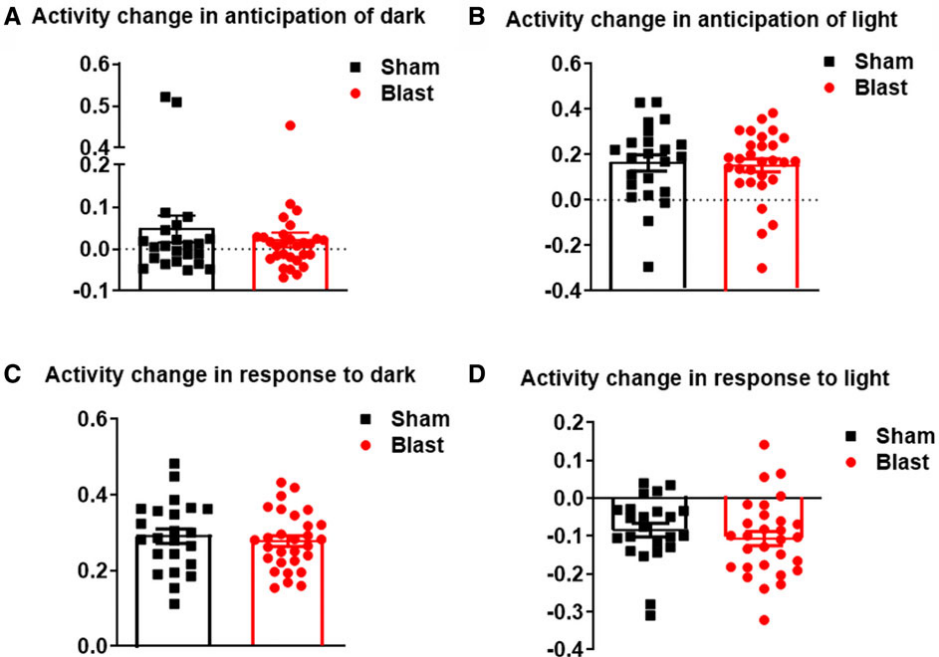
Light/dark phase transitioning behavioral tests in the spontaneous HCM system. Activity changes during the shifts of light and dark phases are analyzed, including (**A**) anticipation of dark, (**B**) anticipation of light, (**C**) in response to dark, and (**D**) in response to light. Data were analyzed using the one-tailed, unpaired Student's *t*-test and are presented as means ± SEM. *n* = 23 sham mice and *n* = 29 LIB-exposed mice. HCM, home-cage monitoring; LIB, low-intensity blast; SEM, standard error of the mean.

Anticipations and responses to the onset of light and dark phases were quantified to capture circadian patterns. Activity changes of anticipation of dark (*p* = 0.2235), and anticipation of light (*p* = 0.4040), as well as in response to dark (*p* = 0.2868), and in response to light (*p* = 0.1995; Fig. 3A–D) showed no significant difference between LIB-exposed and sham mice.

**FIG. 4.**
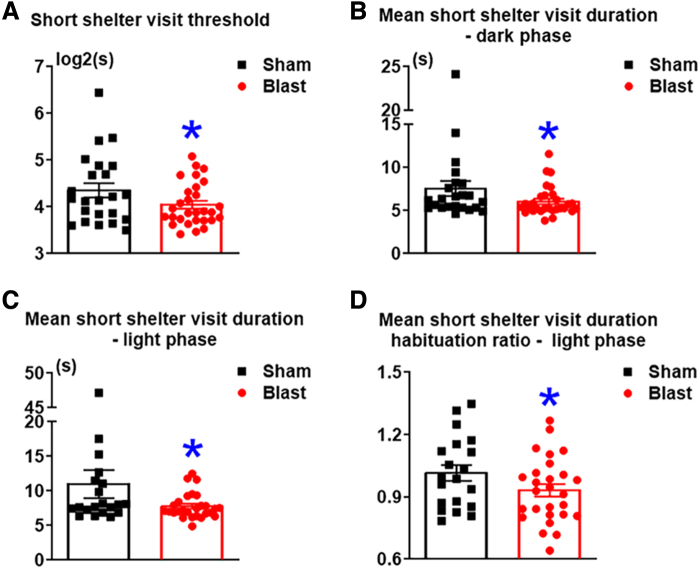
Sheltering behavioral tests in the spontaneous HCM system. Short shelter visits are determined using Gaussian curves representing in-and-out shelter visits during activity bouts, including (**A**) short shelter visit threshold**,** (**B,C**) mean short shelter visit duration during (**B**) light and (**C**) dark phases, as well as (**D**) mean short shelter visit duration habituation ratios during light phases. The threshold of short shelter visits is calculated as log_2_ (seconds). Data were analyzed by the one-tailed, unpaired Student's *t*-test and are presented as means ± SEM. *n* = 23 sham mice and *n* = 29 LIB-exposed mice. HCM, home-cage monitoring; LIB, low-intensity blast; SEM, standard error of the mean.

We further examined the profiles of mouse sheltering behaviors. Shelter visits were distinguished by short, intermediate, and long shelter visits based on the frequency distributions of the shelter visit duration. The short shelter visit threshold defines the upper limit of a short visit.^26^ The short shelter visit threshold was significantly lower in LIB-exposed mice compared to sham controls by 7% (*p* = 0.0349; Fig. 4A). Mean short shelter visit duration was significantly lower in LIB-exposed mice than sham controls in both dark and light phases by 20% (*p* = 0.0457) and 29% (*p* = 0.0399) accordingly (Fig. 4B,C). In addition, habituation ratios of mean short shelter visit duration in light phases were 8% (*p* = 0.0444) lower in LIB-exposed mice compared to sham controls (Fig. 4D). In contrast, duration of long shelter visit showed no significant difference between LIB-exposed mice and sham controls (data not shown).

### Low-intensity blast–exposed mice demonstrated long-term alterations of kinematics in response to an aversive light stimulus assessed in a home-cage environment

**FIG. 5.**
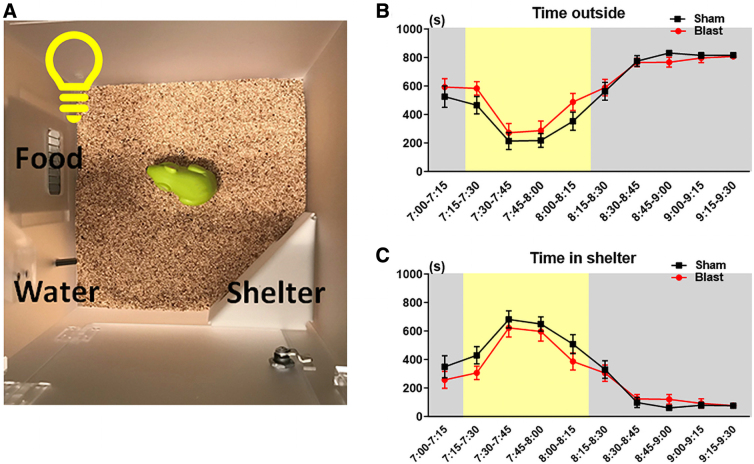
Sheltering behavioral tests in response to spotlight exposure in the HCM system. (**A**) The LightSpot behavioral test is set up the same as the spontaneous behavioral test, except for an aversive spotlight that shines for 1 h during the early dark phases, from 7:15 pm to 8:15 pm. **(B,C)** Sheltering behaviors in response to spotlight exposure are measured with time outside shelter and time in shelter in 15-min bins from 7:00 pm to 9:30 pm. Data were analyzed by two-way repeated-measures ANOVA and Bonferroni multiple comparisons and are presented as means ± SEM. *n* = 20 sham mice and *n* = 28 LIB-exposed mice. ANOVA, analysis of variance; HCM, home-cage monitoring; LIB, low-intensity blast; SEM, standard error of the mean.

The LightSpot test was designed to investigate alterations of animal behaviors in response to aversive stimuli.^27^ Mouse behaviors were assessed in the same home cages as testing the spontaneous behaviors, with spotlight (2000 lux) exposure for 1 h in the early dark phases (Fig. 5A). In a previous study with C57BL/6J mice, spotlight exposure induced a behavioral response showing a decreased proportion of time spent outside the shelter.^27^ Sheltering behaviors were measured in ten 15-min bins, including time spent outside the shelter and sheltering time. Two-way repeated-measures ANOVA showed that there were no significant effects of LIB exposure in time spent outside the shelter (*p* = 0.3343) and sheltering time (*p* = 0.3178; Fig. 5B,C).

**FIG. 6.**
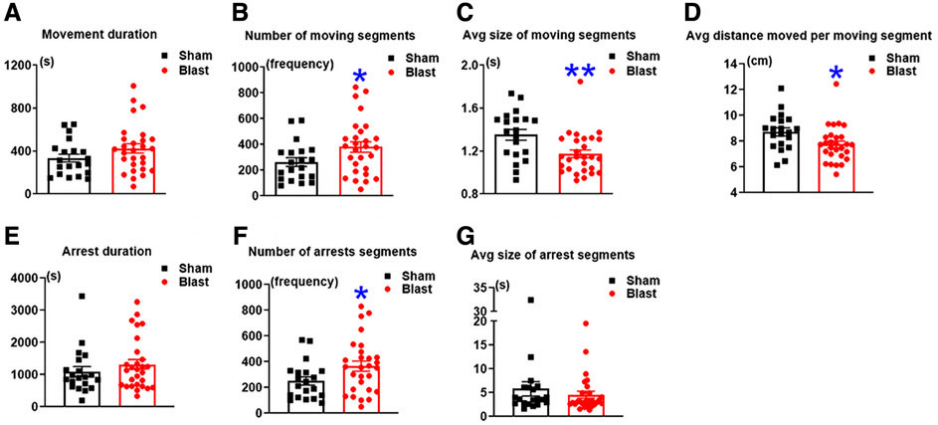
Kinematics in response to spotlight exposure in the HCM system. Parameters of kinematics during the first hour (from 7:00 pm to 8:00 pm) of the LightSpot behavioral test, including (**A**) movement duration, (**B**) number of moving segments, (**C**) average size of moving segments, and (**D**) average distance moved per moving segment, (**E**) arrest duration, (**F**) number of arrest segments, and (**G**) average size of arrest segments. Data were analyzed by the one-tailed, unpaired Student's *t*-test and are presented as means ± SEM. *n* = 20 sham mice and *n* = 28 LIB-exposed mice. HCM, home-cage monitoring; LIB, low-intensity blast; SEM, standard error of the mean.

Kinematics in response to aversive stimuli were further analyzed. During the first hour with spotlight exposure, increased movement duration by 27% was identified in LIB-exposed mice, although this change did not reach statistical significance (*p* = 0.0689). LIB-exposed mice demonstrated significantly higher frequencies of moving segments by 45% (*p* = 0.0204) and significantly decreased average size per movement by 13% (*p* = 0.0026) and average distance per movement by 11% (*p* = 0.0109; Fig. 6A–D). These data suggested that LIB-exposed mice responded to aversive spotlight exposure with anxiety-like movements in higher frequencies, but smaller amplitudes. Significantly higher frequencies of arrest segments by 46% (*p* = 0.0195) were also identified in LIB-exposed mice, whereas no significant changes were found in arrest duration and average size of arrest segments (Fig. 6E–G).

## Discussion

Long-term neurocognitive consequences resulting from blast-induced TBI (bTBI) have posed significant concerns for military service members and veterans.^28^ In a clinical study, 50% of veterans exposed to bTBI experienced anxiety disorders.^29,30^ Anxiety disorders in animal models were characterized by increased arousal, expectancy, autonomic and neuroendocrine activation, as well as a transition from ongoing behaviors to an escape or other defensive behaviors.^31^ Anxiety-like behaviors have been reported 3 months after repetitive, but not single, impact acceleration-induced mTBI in rats.^32,33^ Using animal models of bTBI, studies have identified anxiety-like behaviors in ∼80-kPa blast-exposed rats within 1.5–2.0 months post-injury.^34,35^ Given that neurobehavioral outcomes after blast exposure in animal studies are time- and blast-intensity dependent,^15^ more studies with an advanced behavioral assessment system are needed to investigate long-term neurocognitive consequences induced by open-field LIB.

Anxious states can be characterized as either a temperament, sustained, long-term “trait anxiety” or an acute, ephemeral, fear-induced “state anxiety.”^36^ Conventional tests may not be sufficient and sensitive enough to detect mild behavioral impairments in mice. In this research, we used an automated HCM approach with multiple advantages to assess subtle anxiety-like behaviors in LIB-exposed mice (Table 1).^24,37,38^ With the HCM system, animal behaviors under both natural and challenged conditions can be assessed for distinct spontaneous and affective behaviors.^19,25–27^ Automated, continuous assessments for extended periods in multiple light and dark phases can overcome the limitation of “snapshot” measurements in conventional behavioral assessments.^39^ Moreover, the automated HCM system can limit unavoidable factors such as unintended bias, motivational issues, and circadian shifts during experiments.^24,25^ For testing anxiety-like behaviors in mice, the HCM system has a unique benefit to obtain independent measures of sheltering behaviors and motor function, a major confounding factor for conventional anxiety tests.^40^

**Table 1.** A Brief Comparison of Conventional Behavioral Testing and HCM System

Conventional behavioral testing	HCM system
• Snapshots of behaviors at pre-determined time points during light phases (duration, minutes to hours)	• Assessments of behavioral performance continuously for prolonged periods during multiple light and dark phases (duration, days)
• Human presence and animal handlings affecting behavioral performance	• Automated recordings starting automatically and processing with pre-calibrated experimental protocols without handlings and other human interventions
• Environmental conditions varying across laboratories	• Standardized experimental conditions increasing reproducibility across studies
• Experimental variations generated by individual experimenters	• Automation-based analysis providing rigorousness and reproducibility
• Limited numbers of parameters assessed in one experiment	• Large-scale assessment on multiple parameters of behavioral performance in one experiment (>100 parameters)

HCM, home-cage monitoring.

Kas and colleagues investigated anxiety-like behaviors with chromosome substitution strains-15 (CS-strain 15) mice. Male mice showed reduced movement in the open field, but not in the HCM behavioral tests; CS-strain 15 female mice exhibited altered sheltering behaviors without motor activity abnormality in the HCM tests.^40^ To our knowledge, this is the first study using the automated HCM system to reveal anxiety-like responses on multiple components of the parameters under both natural and challenged conditions in LIB-exposed mice.

In the current study, patterns of spontaneous behavior in LIB-exposed mice and sham controls were evaluated by the HCM system, with >100 behavioral parameters recorded continuously within a 72-h period. Data generated were compared using bioinformatics analysis. Our previous study showed motor dysfunction 1 week after LIB exposure.^1^ However, in this current study, when assessments were made using the HCM system 3 months after LIB exposure, no motor deficits were observed. Moreover, other parameters evaluating general daily performance behaviors, such as activity, arrests, and feeding zone visits, were not significantly different between LIB-exposed mice and sham controls. Effects of light/dark cycles, habituations, and phase transitioning were also not significantly altered in LIB-exposed mice.

Mice have an innate preference for sheltering for a sense of safety in a threatening condition.^40^ In a previous study, anxiety-like behaviors in mice with genetic and pharmacological interventions were estimated by monitoring sheltering behaviors in an HCM system.^40^ To optimize behavioral resolution, sheltering behaviors in this study were further classified according to a previous study.^19,26^ Long shelter visits were used to indicate behaviors of resting or sleeping (for hours), whereas passing through shelters during activity bouts was defined as short shelter visits (for seconds).

In our study, there was no significant difference in long shelter visits between the LIB-exposed mice and sham controls. Interestingly, multiple parameters of short shelter visits relevant to anxiety-like behaviors showed significant differences between LIB-exposed mice and sham controls (Fig. 4). LIB-exposed mice visited their shelters more frequently and for shorter periods of time than sham controls in both dark and light phases. The shorter periods of shelter visit in light phases were more prominent on day 3 compared to day 1 of the experiment in LIB-exposed mice. These results suggest that LIB-exposed mice may hold stable perceptions of environmental stimuli as a threat during activity bouts, whereas sham controls experienced such responses to a lesser degree. This type of performance is consistent as trait anxiety in humans, defined as a tendency to respond with concerns, troubles, and worries to non-threatening situations.^41,42^

Behaviors of LIB-exposed mice were also assessed with a challenged condition by an aversive spotlight under the home-cage environment. Non-injured C57BL/6 mice generally respond to aversive spotlight exposure by increasing sheltering time, followed by decreased sheltering time after spotlight exposure.^27^ In the current study, no significant difference of sheltering behaviors was found in response to aversive spotlight exposure between LIB-exposed mice and sham controls. LIB-exposed mice demonstrated significantly more-frequent activities of movements and arrests and with less amplitudes during spotlight exposure (Fig. 6). The difference among these parameters suggests the presence of anxiety-like behaviors during activity in response to an aversive stimulus in LIB-exposed mice as compared with sham controls. This performance in mice is similar to the state anxiety, which signifies a transitory response evoked by stressful situations and characterized by apprehension, tension, fear, and a heightened autonomic nervous system.^43,44^

It was reported that repeated blast exposure of higher overpressures at 74.5–140.0 kPa could induce long-term PTSD-like behavioral responses, such as exaggerated startle response and anxiety-like behaviors.^45–48^ In the current study, using the highly sensitive HCM system, long-term anxiety-like behaviors were detected in an open-field LIB injury as low as 46.6 kPa. Future studies are warranted to extend the LightSpot test to 72 h to estimate the prolonged effects of aversive stimuli and compare with the 3-day natural, spontaneous activities of the individual mouse in details.

Amygdala reactivity has been shown to play a central role in anxiety disorders.^49^ bTBI has shown neurostructural alterations of amygdala neurons.^50^ Reduced amygdala volume was also observed in veterans with comorbid PTSD/mTBI.^51^ Disruption of hypothalamic/pituitary/adrenal (HPA) homeostasis is another possible biological mechanism that underlies anxiety- and stress-related disorders like PTSD in TBI.^52^ It was reported that LIB-induced TBI may disrupt the paraventricular nucleus, which acts as the central stress regulator of the HPA axis, further inducing hormone dysregulation and psycho- or pathophysiological changes.^53^ However, how the impairments of amygdala and/or HPA induced by blast exposure result in long-term changes in neural circuitry and psychosocial stress are still not clear. Future studies are needed to elucidate the precise mechanisms involved in LIB-induced anxiety disorders.

## Conclusion

In summary, using the highly sensitive HCM system with minimized confounding factors, we were able to detect anxiety-like behaviors on multiple components of the parameters under both natural and challenged conditions in LIB-exposed mice in chronic phase post-injury. This study suggests the need for more sensitive neuropsychological testing and extended long-term assessments of trait anxiety and state anxiety in veterans. Future studies with animal models to elucidate the pathophysiological mechanisms of anxiety disorder involved in LIB exposure are warranted. This study provides insights on alterations in various anxiety-like parameters using a platform model by HCM assessments of diagnostic testing and possible interventions for anxiety disorders for clinical translation on combat-related mTBI.

## Abbreviations Used

ANOVA: analysis of variance
bTBI: blast-induced TBI
CS-strain 15: chromosome substitution strains-15
HCM: home-cage monitoring
HPA: hypothalamic/pituitary/adrenal
LIB: low-intensity blast
mTBI: mild TBI
PTSD: post-traumatic stress disorder
TBI: traumatic brain injury
